# Multistrain Probiotics Alleviate Diarrhea by Modulating Microbiome-Derived Metabolites and Serotonin Pathway

**DOI:** 10.1007/s12602-024-10232-4

**Published:** 2024-03-12

**Authors:** Jin-Ju Jeong, Yoo-Jeong Jin, Raja Ganesan, Hee Jin Park, Byeong Hyun Min, Min Kyo Jeong, Sang Jun Yoon, Mi Ran Choi, Satya Priya Sharma, You Jin Jang, Uigi Min, Jong-Hyun Lim, Kyeong Min Na, Jieun Choi, Sang Hak Han, Young Lim Ham, Do Yup Lee, Byung-Yong Kim, Ki Tae Suk

**Affiliations:** 1 Institute for Liver and Digestive Disease, College of Medicine, Hallym University, Chuncheon, Korea; 2 R&D Center, Chong Kun Dang Healthcare, Seoul, Republic of Korea; 3 Department of Agricultural Biotechnology, Center for Food and Bioconvergence, Research Institute of Agricultural and Life Sciences, Seoul National University, Seoul, Korea; 4 Department of Pathology, College of Medicine, Hallym University, Chuncheon, Republic of Korea; 5 Department of Nursing, Daewon University College Jecheon, Jecheon, Republic of Korea

**Keywords:** Multistrain probiotics, Acetic acid, Microbiome, Metabolites, Cytokines, Diarrhea

## Abstract

Diarrhea, a common gastrointestinal symptom in health problems, is highly associated with gut dysbiosis. The purpose of this study is to demonstrate the effect of multistrain probiotics (Sensi-Biome) on diarrhea from the perspective of the microbiome-neuron axis. Sensi-Biome (*Lactiplantibacillus plantarum*, *Bifidobacterium animalis* subsp. *lactis*, *Lactobacillus acidophilus*, *Streptococcus thermophilus*, *Bifidobacterium bifidum*, and *Lactococcus lactis*) was administered in a 4% acetic acid–induced diarrhea rat model at concentrations of 1 × 10^8^ (G1), 1 × 10^9^ (G2), and 1 × 10^10^ CFU/0.5 mL (G3). Diarrhea-related parameters, inflammation-related cytokines, and stool microbiota analysis by 16S rRNA were evaluated. A targeted and untargeted metabolomics approach was used to analyze the cecum samples using liquid chromatography and orbitrap mass spectrometry. The stool moisture content (*p* < 0.001), intestinal movement rate (*p* < 0.05), and pH (*p* < 0.05) were significantly recovered in G3. Serotonin levels were decreased in the multistrain probiotics groups. The inflammatory cytokines, serotonin, and tryptophan hydroxylase expression were improved in the Sensi-Biome groups. At the phylum level, Sensi-Biome showed the highest relative abundance of *Firmicutes*. Short-chain fatty acids including butyrate, iso-butyrate, propionate, and iso-valeric acid were significantly modified in the Sensi-Biome groups. Equol and oleamide were significantly improved in the multistrain probiotics groups. In conclusion, Sensi-Biome effectively controls diarrhea by modulating metabolites and the serotonin pathway.

**Supplementary Information:**

The online version contains supplementary material available at 10.1007/s12602-024-10232-4.

## Introduction

Diarrhea is a common gastrointestinal disease. Globally, diarrheal disease causes serious complications in approximately 1.5 million children. Diarrhea-dominant irritable bowel syndrome characterized by abdominal pain and altered stool habits is a functional gastrointestinal disorder that affects approximately 5% of the global population [1]. Although medical treatments have improved, diarrhea remains one of the leading causes of health problems and its etiology remains limited [2]. Some microbiotas such as *Clostridium difficile, Escherichia coli*, *Shigella sonnei*, *Shigella flexneri*, and other *Shigella* and *Salmonella* spp*.* may cause diarrhea; the gut microbiota–modulating therapies including probiotics, prebiotics, antibiotics, and microbiota transplantation are being investigated and used. Recently, oral rehydration therapy has been used to reduce mortality from acute diarrhea [3, 4]. In addition to the use of probiotics, phage therapy is also being attempted as a treatment using microorganisms. In this way, for various diseases using microorganisms, it is applied not only to adults but also to infants and children, and shows a positive disease-alleviating effect [5, 6].

*Lactobacillus* spp. are generally probiotics. There are several types of *Lactobacilli*, including *L. acidophilus*, *L. casei*, *L. rhamnosus*, and *L. helveticus*, that are considered successful probiotics. These strains are available for human consumption [7–9]. *L. rhamnosus GG*, *Saccharomyces boulardii*, *Bifidobacterium lactis*, *L. casei*, and *L. paracasei* are significantly effective probiotics for treating diarrhea. Galactosyl-oligosaccharide prebiotics have beneficial effects on gut barrier function. With therapeutic multistain probiotics, *Bifidobacterium longum*, *Bifidobacterium* spp., and *Clostridium perfringens* are more important in preventing and avoiding the recurrence of diarrhea [10]. Diarrheal diseases have been widely associated with low microbial diversity in the human microbiome [11].

Recently, through a literature search, multistain probiotics effective for diarrhea and constipation was developed [12]. We hypothesized that an altered intestinal microbiota composition caused by multistain probiotics can alter the microbiome and metabolites, resulting in decreased intestinal inflammation. The purpose of this study is to demonstrate the effect of multistrain probiotics on diarrhea from the perspective of the microbiome-neuron axis.

## Materials and Methods

### Experimental Animal, Study Design, and Sample Collection

A multistrain probiotics Sensi-Biome containing a mixture of *Lactiplantibacillus plantarum* UALp-05 (Chr. Hansen), *Bifidobacterium animalis* subsp. *lactis* UABla-12 (Chr. Hansen), *Lactobacillus acidophilus* DDS-1 (Chr. Hansen), *Streptococcus thermophilus* CKDB027 (Chong Kun Dang Bio), *Bifidobacterium bifidum* BB-06 (Danisco), and *Lactococcus lactis* MG5125 (Mediogen) was obtained from Chong Kun Dang HealthCare (Seoul, Korea).

All animal experiments were conducted in accordance with the regulations of the Animal Experimental Ethics Committee (IACUC) of Hallym University (Hallym 2021–79). Five-week-old male Sprague–Dawley (SD) rats were purchased from DooYeol Biotech (Seoul, Korea). The SD rats were kept under standard experimental conditions, 20 ± 2 °C; 55 ± 5% humidity; 12-h light/dark cycle; and acclimatized for 7 days.

**Fig. 1:**
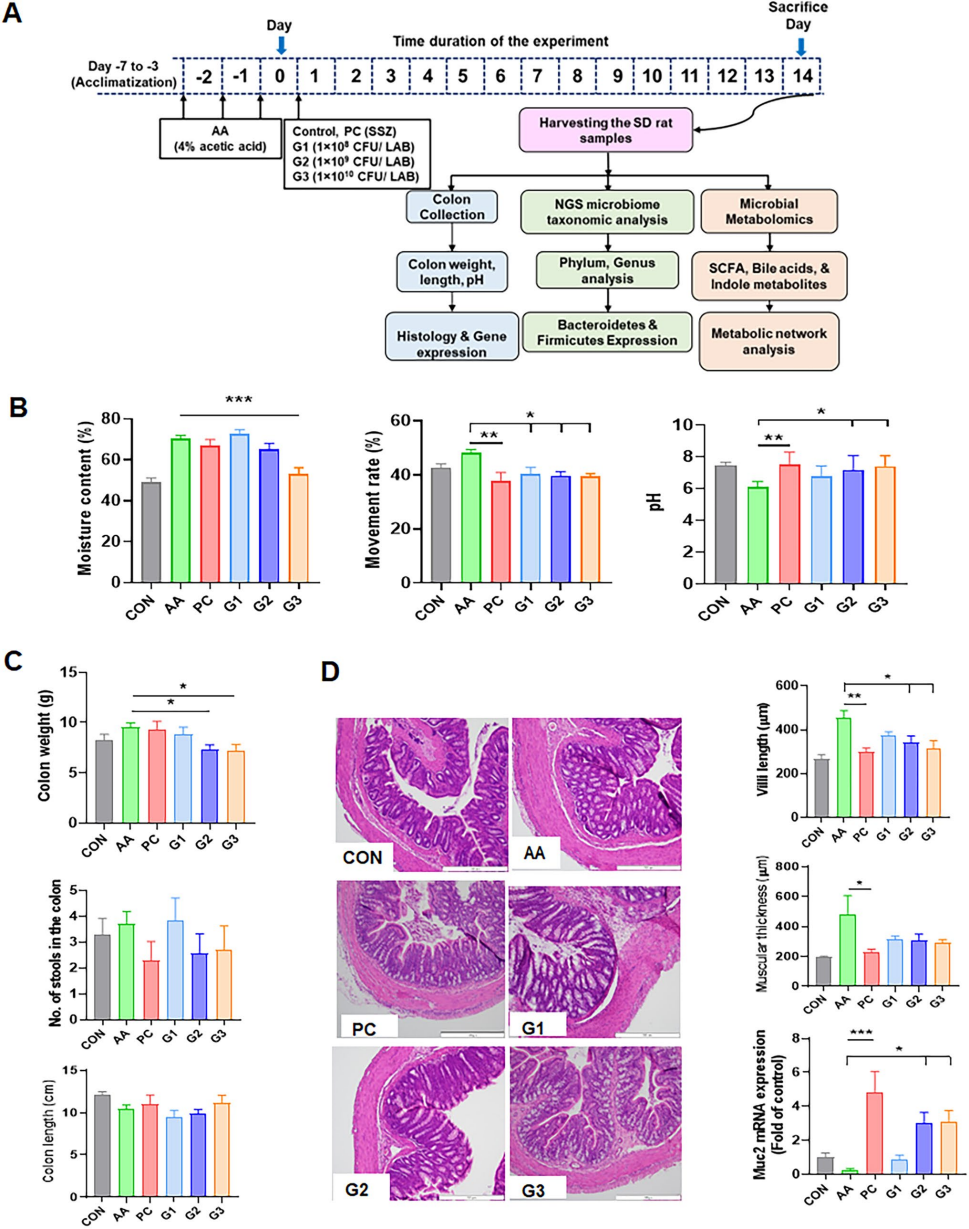
**A** Schematics for the experimental process and 4% acetic acid (AA), SSZ (PC), and multistrain probiotics (G1, G2, and G3) shuttling in SD rats. **B** Stool consistency variation drives species richness in rat. The stool moisture content (%), intestinal movement rate (%), and pH. **C** Total number of stools in colon, colon length (cm), and colon weight (g) was measured. **D** Representative histological sections obtained from the colon tissues, visualized with H&E stain. The images indicate pathological features. The total fibrotic villi length (µm), muscular thickness (µm), and Muc2 mRNA expression of the tunica muscularis of the colon. The gastritis score of gastric tissue from diarrhea infected rats with G1, G2, and G3 multistrain probiotics. Data are represented as mean ± standard error of the mean. *p-value < 0.05; **p-value < 0.01; ***p-value < 0.001

The rats were randomly placed into 6 groups with 7 animals each after a week of acclimatization. In total, 42 SD rats were divided into the following groups: control (CON), acetic acid (negative control: AA), SSZ-positive control (PC: sulfasalazine, 500 mg/kg), and multistrain probiotics (G1: 1 × 10^8^ CFU, G2: 1 × 10^9^ CFU, and G3: 1 × 10^10^ CFU/0.5 mL). Four percentage of acetic acid was injected by intrarectal administration for 3 days for inducing diarrhea. The medicine sulfasalazine (Sigma-Aldrich, St. Louis, MO, USA) was used as a positive control (PC). The experimental method is shown in Fig. 1A with a schematic explanation. SD rats were gavage with multistrain probiotics (G1, G2, and G3) or SSZ (PC) for 14 days. After treatment, feces, colon tissues, and cecum contents were collected and stored at −80 °C before further analysis. The animals were sacrificed by inhalation anesthesia overdose (isoflurane, Aerane; Baxter, Deerfield, IL, USA) at the end of the treatment periods.

### pH, Water Content, and Intestinal Movement Rate

The pH of the diluted fecal samples in distilled water was determined by an Ohause Starter300 pH meter. After incubating the samples at 70 °C for 24 h, the water content was determined by weighing the dry weight. This was done by calculating the difference between the fresh weight and the dry weight.

The effect of intestinal movement rate was measured after fasting 12 h before animal sacrifice. One milliliter of barium sulfate (1.4 g/mL; Daejung Chemicals & Metals Co. Ltd., Siheung-si, Gyeonggi-do, Korea) was orally administered to the experimental animals, and after 30 min, the animals were sacrificed. Finally, we measured the distance of the movement of barium sulfate in the intestine obtained from the animals. The intestinal movement rate was calculated as follows. Intestinal movement rate (%) = distance moved by the barium sulfate (cm)/total intestine length (cm) × 100. At the end of the experiment, the number of stools in the colon and colon length and weight of each animal were measured.

### Real-Time Quantitative Polymerase Chain Reaction

Cytokines, mucin-related gene, and serotonin-related indicators were examined to assess probiotics capacity to control gut immunity. The frozen colon tissue was removed from the refrigerator at −80 °C, weighed at 10 mg, and homogenized using a frozen tissue grinder. For RNA extraction, 50 mg of colon tissue was mixed with 1000 µL of trizol reagent (Life Technologies, Cardisbad, CA, USA). The samples were treated with 200 µL of chloroform (Sigma-Aldrich, St. Louis, MO, USA), incubated for 5 min at room temperature (RT), and centrifuged at 14,000 rpm for 5 min at 4 °C. The RNA pellet was then mixed with 1 mL of 70% cold ethanol and centrifuged at 7500 rpm at 5 min at 4 °C. After collected the supernatant, 100 µL of diethyl pyrocarbonate (DEPC) water was added. All samples were vortexed and dried at RT for 10 min. At wavelengths of 260 nm, RNA quality and quantity were determined. Five micrograms of extracted RNA and 2.5 µL of DEPC water were added to an RT premix (Bioneer, Daejeon, Korea). Specific primer sequences were used to detect mRNAs encoding 5ht1-α, 5ht1-β, sert, Tph2, and Muc2. A 50 µL cDNA was produced using the Mastercycler gradient and utilized as a template for PCR amplification. cDNA was synthesized at 42 °C for 1 h, denatured at 94 °C for 5 min, and denatured at 94 °C for 5 min during the reverse transcription sequence. For PCR, a PCR premix (iNtron, Seongnam, Korea) has been prepared with 10 pg of cDNA, sense primer, and antisense primer with DEPC water as per the manufacturer’s instructions. Amplification was carried out using a master cycler gradient. The gene-specific primer sequences are listed in Table S1.

### ELISA Assay for Serotonin

The levels of serotonin were performed by ELISA kit as previously described (Rat serotonin ELISA kit cat. No. ab133053 Abcam) [13]. In details, samples were thawed on ice and homogenized in DPBS. Thereafter, the samples were homogenized and centrifuged at 1500 × g for 15 min at 4 °C; supernatants were collected, aliquoted, and stored at −20 °C. Concentration of serotonin was measured by ELISA kits according to the manufacturer’s instructions.

### Hematoxylin–Eosin Staining and Immunofluorescence Staining

Specimens were fixed with 10% formalin after extraction and routinely embedded in paraffin, and prepared sections were stained with hematoxylin and eosin. The villi length (µm) and muscular thickness (µm) were assessed. All colon tissues were analyzed by a pathologist (SHH) who was blinded to the experimental conditions. For the immunofluorescence (IF) staining, the colon tissues were cut into 5-µm sections. The tissue sections deparaffinized in xylene and rehydrated in gradient ethanol. The sections were incubated with serotonin primary antibody (1:20, Invitrogen) and TPH1 primary antibody (1:50, Invitrogen). The sections were washed three times with phosphate buffer solution and incubated with goat anti-mouse IgG Alexa Fluor 488 (1:200, ThermoFisher) and goat anti-rabbit IgG Alexa Fluor 633 (1:200, ThermoFisher) secondary antibody for 1 h. Nuclei of the section were stained with DAPI (abcam). Fluorescence was observed with a confocal laser scanning microscope (LSM710, Carl Zeiss). IF intensity was quantified by ImageJ software.

### Microbiome in Fecal Samples

For metagenomic sequencing analysis, genomic DNA was extracted from rat stool and library construction was performed. Briefly, gDNA was extracted using the DNeasy Power Soil kit (Qiagen, Hilden, Germany) according to the manual provided by the manufacturer. To amplify the V3 and V4 regions, sequencing libraries were prepared according to the illumine 16S Metagenomic Sequencing Library protocols. The primers used for PCR amplification were forward (5′-TCGTCGGCAGCGTCAGATGTGTATAAGAGACAGCCTACGGGNGGCWG-CAG and reverse (5′-GTCTCGTGGGCTCGGAGATGTGTATAAGAGACAGGACTACHV-GGGTATCTAATCC). Cycle conditions were 95 °C for 3 min, 25 cycles at 95 °C for 30 s, 55 °C and 72 °C for 30 s, and final extension at 72 °C for 5 min. The PCR product obtained here was purified with AMPure beads (Agencourt Bioscience, Beverly, MA). The purified PCR products were amplified for final library construction using NexteraXT Indexed Primer. All PCR conditions were the same described above, except that the number of cycles was 10 times. After purification of the PCR products, they were quantified by qPCR using KAPA Library Quantification kits for Illumine Sequencing platforms, and qualified with TapeStation D1000 ScreenTape (Agilent Technologies, Waldbronn, Germany). Then, sequencing was performed using the MiSeq platform (Illumina, San Diego, USA); sequence analysis of the samples was conducted by the Macrogen Inc. (Macrogen, Seoul, Korea).

For amplicon sequence variants (ASV) analysis, the Cutadapt (v3.2) program was used to remove the sequencing adapter sequence and the F/R primer sequence of the target gene region [14]. Error correction of the amplicon sequencing process was performed using the DADA2 (v1.18.0) package of R (v4.0.3) [15]. After combining the corrected paired-end sequences into one sequence, the chimera was removed. Using QIIME (v1.9), subsampling was applied and normalized based on the number of reads of the sample with the minimum number of reads among all samples, and the microbial community was compared and analyzed [16].

### Sample Preparation for Metabolic Profiling

The metabolomic profiles of mouse cecum were obtained through short-chain fatty acid–targeted and untargeted liquid chromatography-mass spectrometry (LC–MS) methods. One hundred microliters of each cecal sample from an individual mouse was collected in 2-mL Eppendorf tube. Subsequently, 1700 µL of 4 °C refrigerated extraction solvent (MeOH:IPA:D.W. = 3:3:2) was added to each Eppendorf tube [17]. Samples were homogenized by a M400 homogenizer (Haan, Germany, Letsch GmbH & Co.) The mixed samples were then sonicated for 10 min under ice-cold condition (DAIHAN, Korea). After the centrifugation (13,200 rpm, 10 min, 4 °C, Eppendorf centrifuge 5415 R), 40 µL of supernatant from each sample was aliquoted and transferred to 2-mL Eppendorf tube, which was then stored in a −80 °C deep freezer until short-chain fatty acid derivatization [18]. Simultaneously, 500 µL of the supernatant for untargeted mass spectrometry was transferred to 1.5-mL Eppendorf tube, followed by evaporation using a speed vacuum concentrator (ScanVac, Korea). The concentrated residue was stored in −80 °C deep freezer until the untargeted LC–MS analysis.

#### Method 1: Untargeted Metabolite Profiling of LC-Orbitrap MS

The dried residue was reconstituted using 250 µL of 70% ACN (v/v). Following a 10-min ice-cold sonication, the samples were filtered through PTFE filter with centrifugation (4 °C, 11,000 rpm, 1 min) [19]. To target bile acids and indoles, a set of 10 bile acid standards and 5 indole standards were reconstituted using 70% ACN to achieve 100 µM each. Subsequently, 5 µL of each sample was injected for analysis in each sequence.

The Ultimate-3000 UPLC (Thermo, Waltham, MA, USA) with Acquity UPLC BEH C18 column (2.1X100 mm, Water, Milford, MA, USA) was used for chromatographical separation. The LC system was controlled using Chromeleon 6.8 operation software (Dionex, CA, USA). The mobile phase for analysis consisted of solvent A (D.W.) and solvent B (ACN) both containing 0.1% formic acid (v/v). Flow rate was set at 0.3 mL/min, and the gradient elution was set up as following: 0–0.5 min, 0.5% B; 0.5–10 min, 0.5–80% B; 10–10.1 min, 80–99.5% B; 10.1–12 min, 99.5% B; 12–12.1 min, 99.5–0.5% B; 12.1–15 min, 0.5% B. The mass spectrometric analysis was conducted using Q-ExactiveTM Plus Hybrid Quadrople-Orbitrap Mass Spectrometer (Thermo, Waltham, MA, USA), and data acquisition and pre-processing were performed using Thermo Scientific™ Xcalibur 4.0. The RAW data files obtained after sequencing were processed by using Compound Discoverer software (version 3.2, Thermo Fisher Scientific, San José). The data processing workflow encompassed the following steps: Select Spectra, Filter By Scan Event, Align Retention Times, Detect Compounds, Group Compounds, Assign Compound Annotations, Predict Compositions, Fill Gaps, and Search mzCloud. The data processing for the ten bile acids and five indoles was conducted using Tracefinder software (version 4.1, Thermo Fisher Scientific, San José, CA, USA). The metabolites were validated with reference compounds.

#### Method 2: SCFAs-Targeted Analysis with LC-Orbitrap MS

The 40 µL of the aliquoted supernatant and six 100 uM short-chain fatty acid (SCFA) standard compounds were each mixed with 20 µL of 200 mM 3-nitrophenylhydrazine hydrochloride in 70% ACN (v/v) and 20 µL of a 120 mM 1-ethyl-3-(3-dimethylaminopropyl)carbodiimide hydrochloride in 6% pyridine solution [19]. The resulting mixture was incubated for 30 min at 40 °C and then diluted with 1.92 mL of 70% ACN. The derivatives were chromatographically separated using the same column as employed in the untargeted profiling, under the control of Ultimate-3000 UPLC (Thermo, Waltham, MA, USA). The mobile phase composition and flow rate were consistent with the untargeted profiling method. The gradient of LC elution was set up as follows: 0–2 min, 15% B; 2–11 min, 15–55% B; 11–11.1%, 55–100% B; 11.1–12 min, 100% B; 12–12.1 min, 100–15% B; 12.1–15 min, 15% B. An injection volume was 2 µL. Mass spectra were acquired using the Q-Exactive Plus Orbitrap (Thermo Fisher Scientific, Waltham) equipped with an electrospray ionization (ESI) interface in negative ionization. The system was controlled using Xcalibur 4.0 and Q-Exactive Tune software. The acquired RAW data were processed by Tracefinder software.

### QuantSeq 3′mRNA-Sequencing

Total RNA sequencing analysis extracted from mouse liver tissue was performed by eBiogen Inc. (Seoul, South Korea). Gene ontology (GO), differentially expressed genes (DEG), clustering heatmap, and principal component analysis were performed using ExDEGA (Excel-based differentially expressed gene analysis) tool and ExDEGA Graphic Plus provided by eBiogen Inc. (Seoul, South Korea). Gene correlation analysis based on the Protein–Protein Interaction database was performed using the Cytoscape STRING tool (https://string-db.org/).

### Statistical Analysis for Data Integration

Metabolite normalization was verified. Logarithmic transformation was applied before analyzing metabolic expression, metabolic networking, and data interpretation. In all data, *p* values were calculated using an independent *T*-test, Kruskal–Wallis *H*-test, Wilcox, and ANOVA test (*p* < 0.05). GraphPad Prism v8.0 (GraphPad Software, Inc., San Diego, CA, USA) was utilized to visualize and assemble the figs. The mean and standard error deviation (mean ± SE) were represented in all data. For the statistical analyses conducted on LC–MS data, significant differences between two groups were determined by the Mann–Whitney *U* test. The dot plot was visualized using GraphPad Prism ver. 9 (GraphPad Software Inc., San Diego, CA, USA). SIMCA (Ver 17.0.0.24543, Umetrics, Umea Sweden) was applied to perform multivariate statistics, including principal component analysis (PCA), partial least square discriminant analysis (PLS-DA), and VIP score. The metabolomic network structure was constructed based on Tanimoto score and Kyoto encyclopedia of genes and genomes (KEGG) reaction pairs and visualized using Cytoscape ver. 3.9.1 [20].

## Results

### Improvement of Diarrhea-Related Indicators in Stools and Colon of Rats

The experimental scheme is shown in Fig. 1A. Multistrain probiotics changes in the colon and stool were confirmed by diarrhea induction. Stool moisture content (*p* < 0.001), intestinal movement rate (*p* < 0.05), and pH (*p* < 0.05) were significantly recovered in G3 compared with AA (negative control). In addition, the intestinal movement rate and pH of PC (positive control) were significantly improved compared to those of AA. The pH of G2 was also significantly increased compared to AA (Fig. 1B). Colon weight was significantly improved in the G2 and G3 groups. Although the total stools in the colon and colon length in the experimental rats did not exhibit significant differences, improvement tendencies in the PC and multistrain probiotics groups were observed compared to the AA group (Fig. 1C).

In Fig. 1D, the histopathological results of colons are shown. Evaluation of the colons demonstrated that the villi were significantly lengthened in AA (452.5 µm ± 34.9) compared with the control (267.5 µm ± 19.2). In PC (299.3 µm ± 17.5) and G3 (316.9 µm ± 33.5), the villi were shortened compared to AA. G1 (371.9 µm ± 18.8) and G2 (340.7 µm ± 30.9) also showed a similar trend to G3. PC (226.9 µm ± 21.3) also significantly reduced muscular thickness compared with AA (482.2 µm ± 123.4).

**Fig. 2:**
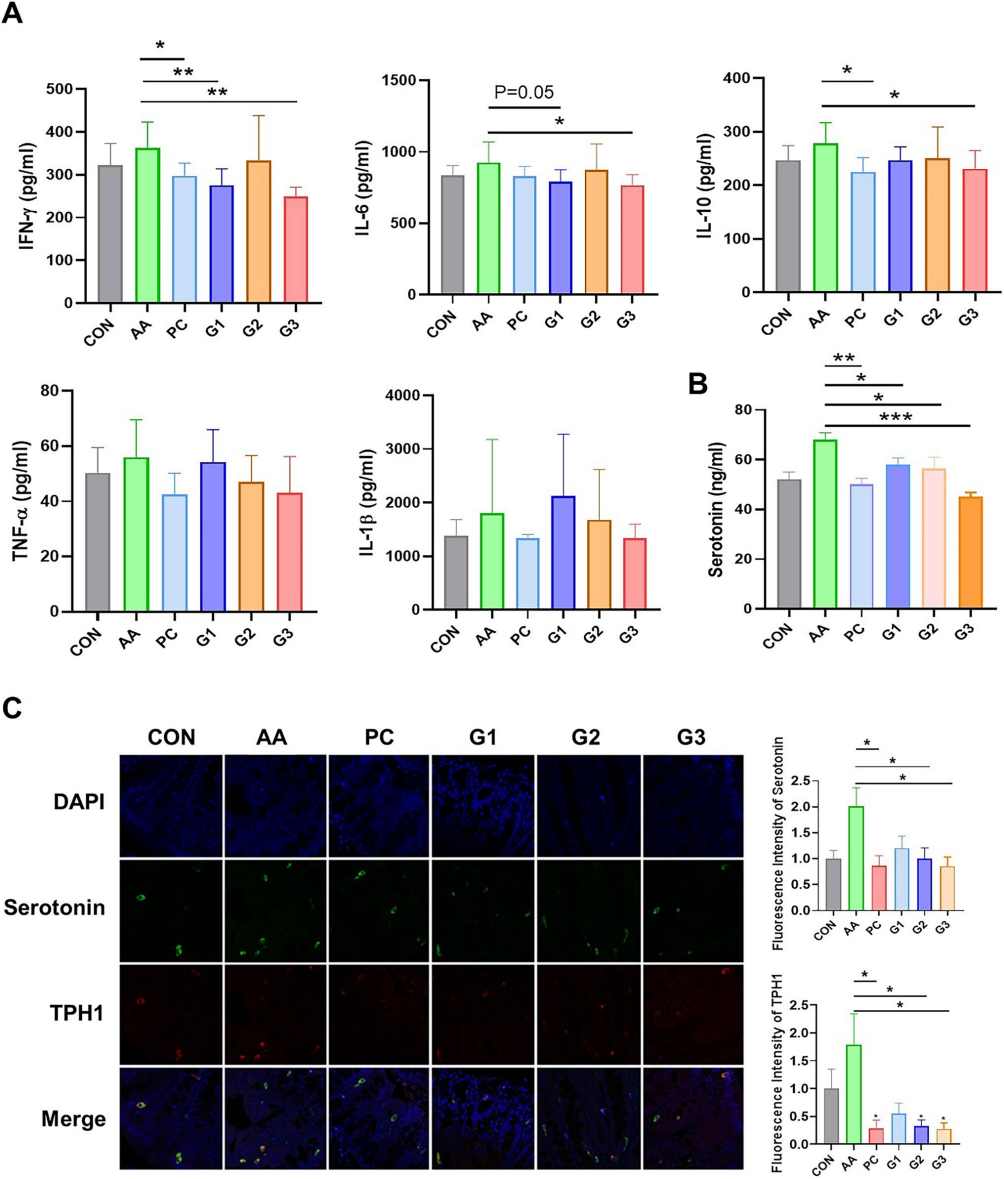
Cytokines and serotonin were measured. **A** Amounts of TNF- α, IFN-δ, IL-1β, IL-6, and IL-10 were monitored by multistain probiotics. The expression level in colon cells subjected and assessed after the treatment of G1, G2, and G3 multistrain probiotics. The probiotics effects and validation has done by ELISA. **B** Serotonin level. **C** Immunofluorescence staining of serotonin and TPH1 gene expression in diarrhea. Fluorescence intensity of serotonin and TPH1 expression. *p < 0.05 and **p < 0.01. Statistical comparisons were performed using mean ± standard error of the mean

In addition, the mRNA expression of Muc2 was significantly increased in PC (4.8 ± 1.2), G2 (3.0 ± 0.6), and G3 (3.1 ± 0.6) compared to AA (0.3 ± 0.1). The constitutive expression of Muc2 mRNA genes may be necessary for normal epithelial cell function during inflammation. Likewise, the ELISA of the inflammatory factors IFN-γ and IL-6 was significantly decreased in G1 and G3 (Figs. 1D and 2A).

### Changes in Expression of Serotonin by Diarrhea Induction and Probiotics

It is known that the secretion of serotonin is related to the induction of diarrhea [21]. Therefore, IF staining and ELISA were conducted to quantify serotonin and related gene levels in colon tissues. IF staining labeled serotonin (green) and TPH1 (red) (Fig. 2B). In the diarrhea induction group AA, the fluorescence intensities of serotonin and TPH1 were 2.0 ± 0.4 and 1.8 ± 0.6, respectively. Compared to AA, PC, G2, and G3 were significantly decreased in the fluorescence intensity of serotonin. The TPH1 was also significantly decreased in both groups (PC: 0.3 ± 0.2; G3: 0.3 ± 0.1) and G2 (0.3 ± 0.1). In addition, the serotonin expression level in the colon was significantly increased in AA (64.8 ± 1.7) compared to CON (54.3 ± 3.6). As in the IF of serotonin, serotonin levels (ng/mL) in the colon were significantly decreased in PC (*p* = 0.0016), G1, G2, and G3 (*p* < 0.0001). The serotonin levels were 49.9 ± 4.4, 58.1 ± 2.8, 54.6 ± 5.4, and 47.8 ± 1.9 in PC, G1, G2, and G3, respectively (Fig. 2B). These data supported that improvement of diarrhea by multistrain probiotics could be related to adjustment of serotonin levels in the colon.

At the mRNA level, serotonin-related factors (receptor, precursor, and transporter), including 5ht1α (*p* < 0.001 in G3), 5ht1β (*p* < 0.01 in G3 and PC), Sert (*p* < 0.01 in G3 and PC), and Tph2 (*p* < 0.01 in G3; *p* < 0.05 in PC), were significantly increased in PC and G3 compared to AA (Fig. S3).

### Changes in Microbiome by Diarrhea Induction and Multistrain Probiotics

**Fig. 3:**
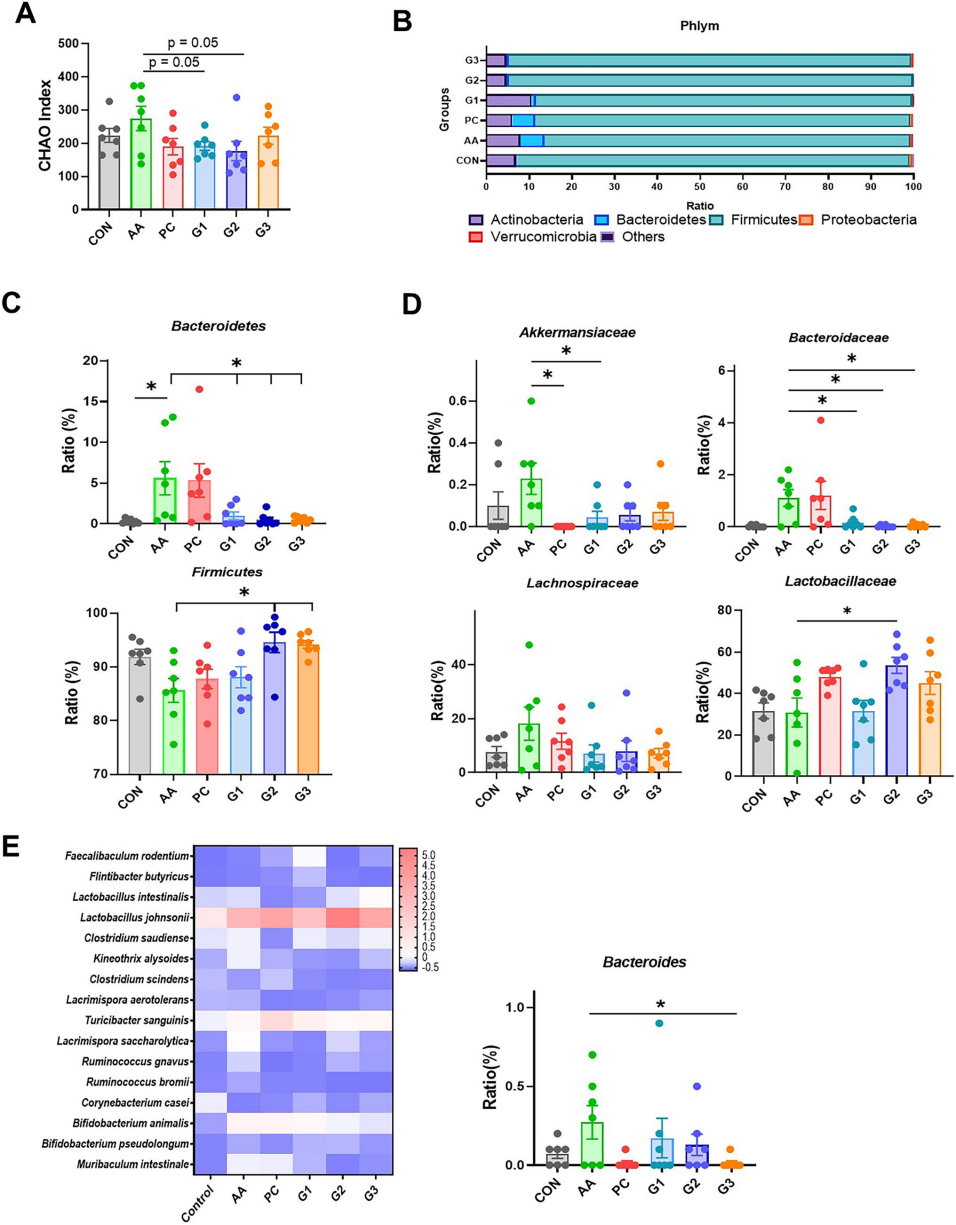
**A** Relative bacterial diversity index analysis at CHAO. Microbial indexes were calculated to assess the intestinal microbial community. **B** Phylum distribution of the number of species that are either elevated or depleted in diarrhea stages compared to the CON. **C** The phylum levels in *Bacteroidetes* and *Firmicutes*. **D** The bacterial ratio (%) of *Akkermansiaceae*, *bacteroidaceae*, *Lachnospiraceae*, and *Lactobacillaceae* shows the variability. **E** Genus-level bacteria were found between the multistrain probiotics. The *Bacteroides* ratio expression was noted. The number of observed changes was very high; most species demonstrated alterations that are specific to diarrhea. Statistical analysis was performed by the Kruskal–Wallis *H*-test. *p < 0.05. Statistical comparisons were performed using mean ± standard error of the mean

To assess gut microbiota alpha diversity, the CHAO, Shannon, and Simpson indicators were calculated based on next-generation sequencing data. CHAO measures the number of species present in a sample, Shannon measures species richness and evenness, and Simpson measures species dominance. In the CHAO, Shannon, and Simpson indices, significant differences were not observed between all the groups (Figs. 3A and S1A). These data showed that dysbiosis did not occur after chemical or multistrain probiotics treatment.

At the phylum level, significant ratio changes in *Bacteroidetes* (*p* < 0.05 in CON vs. AA) and *Firmicutes* (*p* < 0.05 in G2 and G3 vs. AA) were observed. Multistain probiotics had the highest relative abundance of *Firmicutes* and *Bacteroidetes*. *Bacteroidetes* was significantly increased in the diarrhea-inducing AA group compared with the control (Fig. 3B and C). On the other hand, it tended to decrease in all multistrain probiotics groups (Fig. 3D).

At the family level, the abundance of *Akkermansiaceae* tended to increase in AA compared to all other groups. It was significantly reduced in PC compared with AA. A similar tendency was observed in *Lachnospiraceae*. *Bacteroideaceae*, which belongs to the phylum *Bacteroidetes*, was significantly increased in PC compared with CON, G1, G2, and G3. It was significantly decreased in G2 and G3 compared to PC. In G2, *Lactobacillaceae* was significantly increased compared to AA. The observed *p* values were < 0.05, which showed statistically significant differences at the family level. The abundance of *Bacteroides* was increased in AA and recovered in PC and G3. However, significant differences were not observed in G1 and G2 compared with AA (Fig. 3E). *Romboutsia timonensis* and *Clostridium saudiense* were increased in G1 and G3 compared to PC. *Lactobacillus intestinalis* was significantly increased in G3 compared to PC. Increasing tendency in both species was observed in all other probiotics groups. Otherwise, *Vallitalea pronyensis* was significantly reduced in G2 and G3 compared to PC (Fig. S2).

### Metabolites Change by Induction of Diarrhea and Multistrain Probiotics

**Fig. 4:**
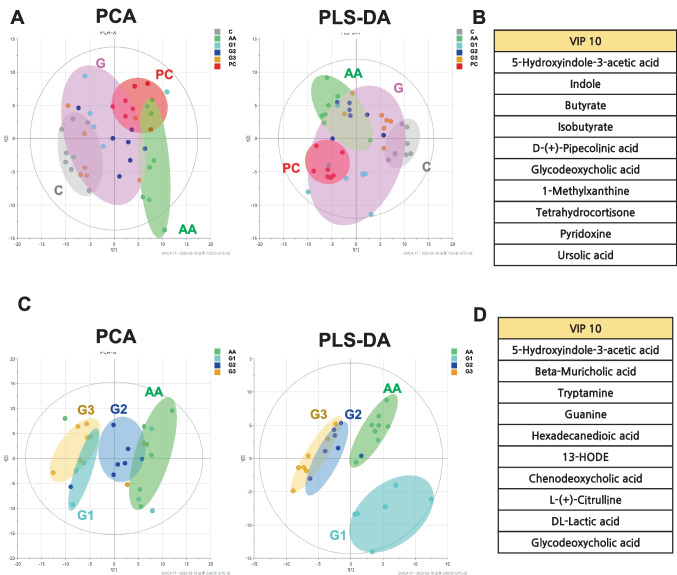
Metabolomic characteristics of the cecum between the multistrain probiotics treated SD rats. **A** PCA score plot of multistrain probiotics (R^2^ = 0.296 and Q^2^ = 0.173). PLS-DA score plot of multistrain probiotics (R^2^ = 0.548 and Q^2^ = 0.168). Control, PC, G, and AA. **B** TOP ranking metabolites. **C** PCA score plot of multistrain probiotics. PLS-DA score plot of multistrain probiotics. G1, G2, G3, and AA. (D) TOP ranking metabolites

A total of 250 metabolite profiles of fecal samples (mass spectral features with unique MS/MS fragmentation patterns) were observed with untargeted analysis and SCFAs-targeted analysis. The compound ontology classified the metabolites, with 56% of them categorized as superclass lipids and organic acids. The sub-classes of the major group were as follows: carboxylic acids, fatty acyls, and steroids accounted for 57, 47, and 22 compounds, respectively (Fig. S4, Table S2). Using PCA (principal component analysis) and PLS-DA (partial least squares discriminant analysis) models, the metabolic characteristics of fecal metabolites in CON, AA, PC, G1, G2, and G3 were predicted. The metabolic difference between the groups can be seen in Fig. 4A: PCA (R2X = 0.296; Q2 = 0.173) and Fig. 4B: PLS-DA (R2X = 0.272; R2Y = 0.276; Q2 = 0.168). Both PCA and PLS-DA models showed that AA was clearly separated from CON. PC also clearly separated from CON in both models. In addition, it partially overlapped with AA in the PCA model. Both analyses demonstrated that multistrain probiotics treatment approximated the control (Fig. 4A and B). Without the PC and CON groups, both the PCA and PLS-DA models showed that AA was clearly separated from the multistrain probiotics groups (Fig. 4C and D). To determine that the metabolites contributed to the model, we prioritized the metabolites using variable importance projection (VIP) based on the PLS-DA model (Fig. S5).

**Fig. 5:**
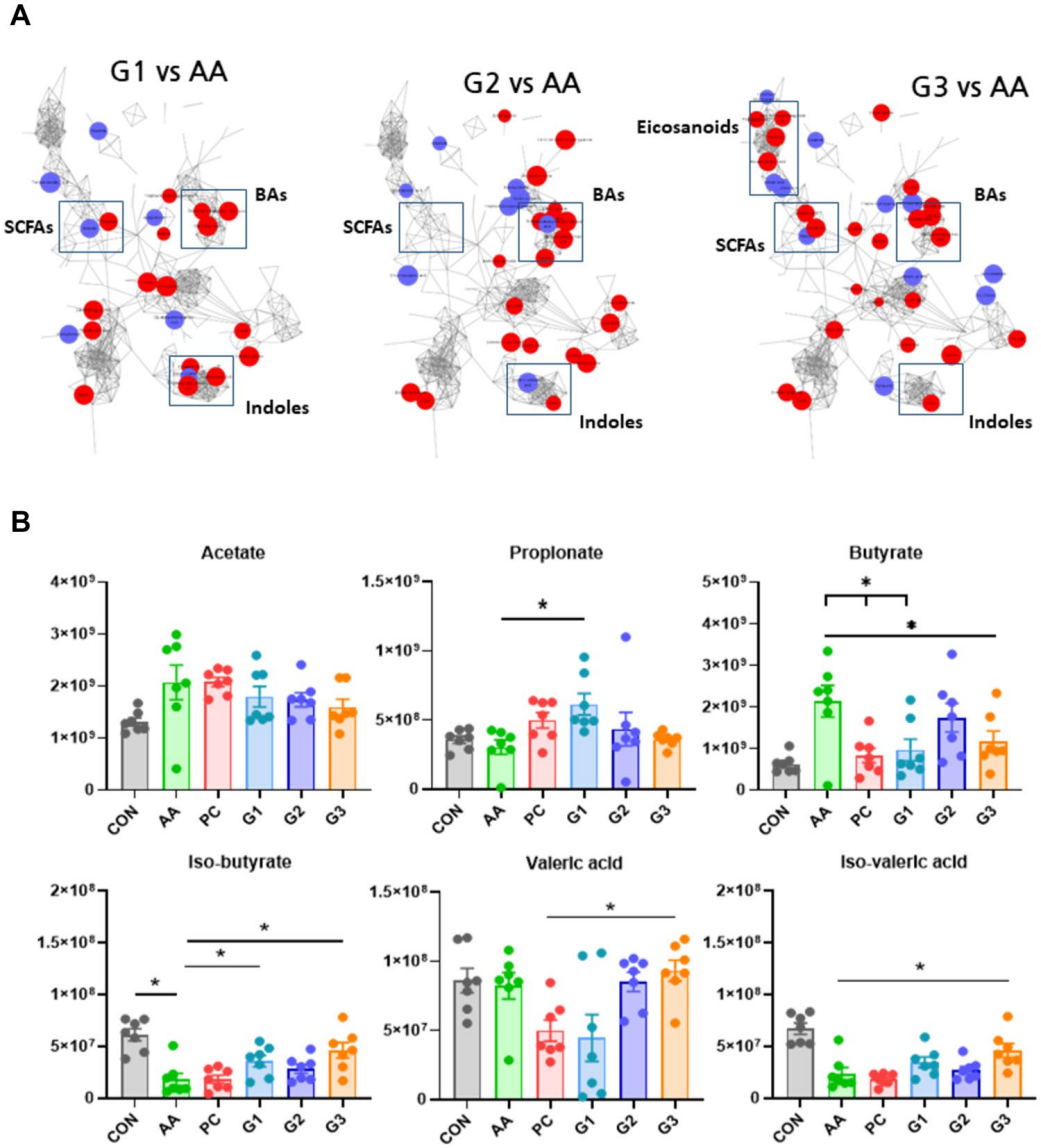
Metabolomic characteristics. **A** A stratified integrated metabolic network was constructed with chemical structural similarity (Tanimoto score) and KEGG reaction pairs. **B** Concentrations of total SCFAs. The six representative SCFAs such as acetate, butyrate, iso-butyrate, propionate, valeric acid, and iso-valeric acid. *p < 0.05. Statistical comparisons were performed using mean ± standard error of the mean

To effectively identify the function of metabolites, a stratified integrated metabolic network was constructed with chemical structural similarity (Tanimoto score) and KEGG reaction pairs to confirm the pattern between the disease group and each probiotic-treated group (G1, G2, and G3). In the G1/AA analysis, propionate, 5-hydroxyindole3-acetic acid, 3-indoleacetic acid, deoxycholic acid, glycocholic acid, and beta-muricholic acid were increased (Fig. 5A). Indole compounds were commonly increased.

Research on SCFAs in diarrhea has been ongoing for over 2 decades. There was a significant difference in the profile of SCFAs such as propionate, butyrate, iso-butyrate, valeric acid, and iso-valeric acid between the groups. Although acetate did not show a significant difference between the groups, it tended to increase in AA and PC compared to CON. Propionate was significantly increased in G1 compared to AA. In the diarrhea induction group AA, significantly increased levels of butyrate and decreased levels of iso-butyrate and iso-valeric acid were observed compared to the normal group CON. Increased butyrate was significantly decreased in PC, G1, and G3. The increase or decrease in SCFAs seen with multistrain probiotics treatment was not observed in PC except with butyrate. Moreover, valeric acid was significantly increased in G3 compared to PC. Iso-butyrate and iso-valeric acid were significantly increased in G1 and G3 compared with AA (Fig. 5B).

**Fig. 6:**
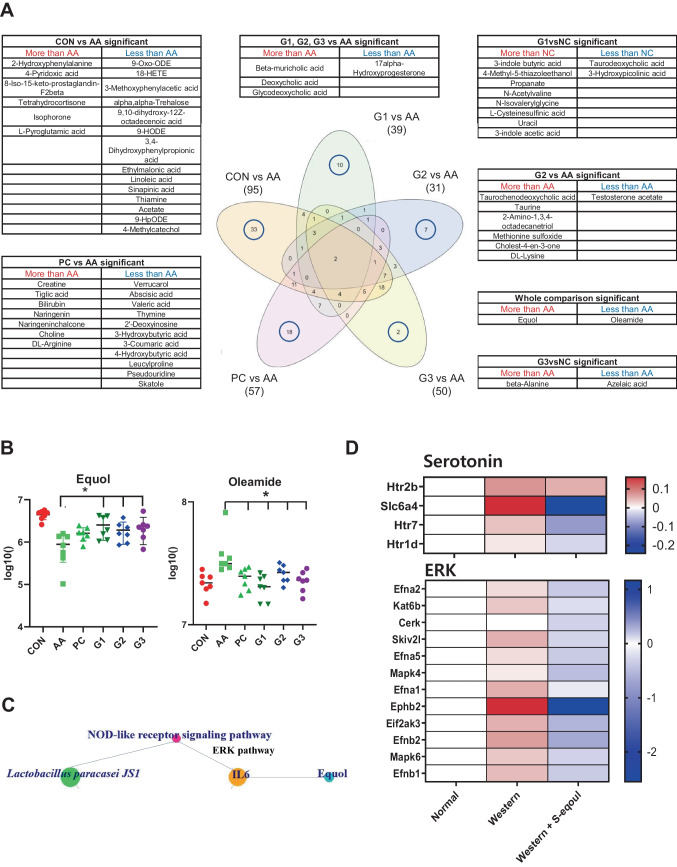
**A** Venn diagram showing the significant metabolites predicted multistain probiotics. Tables show significant differences in metabolites between diarrhea groups. Red and blue metabolites indicate increases or decreases, respectively. **B** Equol and oleamide. **C** Network pharmacology. **D** Heatmap for equol effect. QuantSeq 3′mRNA-sequencing. *p < 0.05. Statistical comparisons were performed using mean ± standard error of the mean

Based on univariate statistics (Mann–Whitney *U* test), significant compounds were identified when the CON, PC, and multistrain probiotic groups were compared with the AA group. All comparisons showed an increase or decrease compared to the AA, with compounds marked in red representing increases and compounds marked in blue representing decreases (Fig. 6A).

### Metabolites Change by Diarrhea Induction and Multistrain Probiotics

In the comparison analysis, equol, beta-muricholic acid, glycodeoxycholic acid, and deoxycholic acid were commonly increased in the G1/AA, G2/AA, and G3/AA groups. Oleamide and 17alpha-hydroxyprogesterone were decreased in the G1/AA, G2/AA, and G3/AA groups. In addition, equol and oleamide showed common patterns of change (Fig. 6B).

In the network pharmacology analysis, equol was related to the IL 6 and ERK pathways (Fig. 6C and D). To investigate the effect of equol, a Western diet animal model was created. Administration of equol confirmed the decrease in serotonin and ERK-related factors.

## Discussion

In this study, we assessed the modulation effect of multistrain probiotic mixtures on the rat gut microbiome with metagenomics data. Our results revealed that multistrain probiotics exerted a distinctive effect on reshaping the gut community compared with that of nonprobiotic treatments. Multistrain probiotic treatment was more effective than AA, and PC treatment improved microbial diversity and diversified the microbial community structure. As a result of our experiment, multistrain probiotics exerted a unique effect on modulating the gut microbiome rather than simply combining the effects of the components of multistrain probiotics.

In this study, multistrain probiotics showed the effect of regulating the secretion of serotonin. SCFA and butyrate induce the release of serotonin through a cholinergic mechanism [22, 23]. SCFA levels decline from the proximal to distal colon, and pH rises from the cecum to the rectum. Reduced mucosal bicarbonate secretion, increased mucosal content, bacterial lactate production, and SCFAs metabolism are known to be factors affecting colonic acidification in patients with inflammatory bowel disease and ulcerative colitis [24, 25]. Among these factors, lactate (lactic acid) is one of the VIP score Top 10 features that influenced group classification in multivariate analysis for disease groups and other groups (Fig. 4). Therefore, lactate could influence the level of pH value. SCFAs and BAs (deoxycholic acid and beta-muricholic acid) have a significant biological role in diarrhea. There is evidence that gut metabolites such as butyrate can inhibit AMPK expression and gluconeogenesis [26]. In our results, butyrate was significantly decreased in multistrain probiotics groups. Taken together, these results suggest that multistrain probiotics reduce butyrate production in gut microbiotas, thereby reducing serotonin secretion and controlling intestinal motility.

Probiotics affect equol levels and equol-producing bacteria in the gut. Equol is the most potent soy isoflavone metabolite and is produced by gut microbiotas [27, 28]. The factors inhibiting equol production enhance the clinical effectiveness of diarrhea. In this study, equol was commonly increased metabolite. Multistrain probiotics contain *L. plantarum*, *B. animalis* subsp. *lactis*, and *L. acidophilus*, and these microbiotas are related to the production of equol [27]. Our pharmacologic analysis showed equol was associated with *Lactobacillus*, ERK pathway, and IL-6. QuantSeq 3′mRNA-sequencing data showed equal effectively control ERK pathway. According to the results of the study, multistrain probiotics control diarrhea by secreting equol and regulating IL-6 relating ERK pathway.

Our study found that both *Firmicutes* and *Bacteroidetes* increased in multistrain probiotics, despite significant interindividual variation. *Firmicutes* and *Bacteroidetes* have been reported to increase and decrease diarrhea, respectively [29]. Diarrhea treated by multistrain probiotics is regarded as osmotic diarrhea [30]. A multistrain probiotics diet has often been used for inducing persistent diarrhea. *Akkermansiaceae*, *Bacteroidaceae*, and *Lactobacillaceae* were chosen as therapeutic biomarkers to induce an anti-diarrhea experiment. *Firmicutes* and *Bacteroidetes* were measured in the diarrheal microbiome [31, 32]. From the results, the acetic acid–induced diarrhea, the *Akkermansiaceae*, *Bacteroidaceae*, and *Lactobacillaceae* have been changed; this is not a phenotype caused by probiotics, but rather one associated with the diarrheal disease.

The microbiome at the phylum and genus levels is known to have a positive effect on gut health, and it was hypothesized that their presence would help reduce the severity of diarrhea symptoms by multistrain probiotics [33]. The total number of stools per day and an increase in the average stool consistency score in the colon.

Several bacterial strains, such as *Morganella morganii* (NCIMB10466), *Klebsiella pneumoniae* (NCIMB673), *Lactococcus lactis subsp. cremoris* (MG1363), *Lactococcus lactis subsp. lactis* (IL1403), *Lactobacillus plantarum* (FI8595), and *Streptococcus thermophilus* (NCFB2392), produce serotonin or 5-hydroxytryptamine (5-HT) [34–36]. In addition, spore-forming gut bacteria raise host serotonin levels. The indole pathway releases serotonin from enterochromaffin cells in the gut, which increases its ability to inhibit brain cells. *Lactobacillus* and *Clostridium* produce tryptophan that is catabolized by intestinal homeostasis.

Apoptosis and inflammation are induced by excessive ROS produced by multistrain probiotics, which activate the TNF-α, NF-β, and JNK signaling pathways. As a result, multistrain probiotics have low chronic inflammation [37, 38]. According to inflammatory cytokine alterations, SD rats were hypertrophic, and the levels of proinflammatory cytokines were decreased, while the anti-inflammatory factors IL-10 and IL-6 were not significantly modified [39]. In addition to being anti-inflammatory cytokines, IL-10 and IL-4 also inhibit the secretion of proinflammatory cytokines such as IL-2, IFN-a, TNF-a, IL-1, IL-6, and IL-8. For example, macrophages, T cells, and other immune cells are regulated to suppress the immune response [40, 41]. LF-CQPC07 significantly reduces inflammatory signals. The inflammatory factors TNF-α, IL-6, IL-1β, and IFN-γ play a role in low-grade inflammation by multistrain probiotics. The levels of the anti-inflammatory factors IL-10 and IL-4 are increased. The inhibition of chronic low-grade inflammation is associated with obesity [42]. The TPH1 gene catalyzes the biosynthesis of serotonin, which is an important hormone and neurotransmitter.

The multistrain probiotic-induced microbial mechanisms of indole compounds such as 3-indole acetic acid and 3-indole propionic acid exhibited metabolic changes. Indole derivatives are manufactured by numerous bacterial strains involved in tryptophan metabolism. Multistrain probiotics applied to tryptophan catalyzers remain undiscovered [43]. Tryptophan metabolism also affects host metabolism through its metabolites. Metabolic diseases alter these pathways [44]. *Ruminococcus gnavus* uses a tryptophan decarboxylase enzyme to transform tryptophan into tryptamine [45]. This serotonin-induced inhibition allows for improved communication between different brain cells, leading to better overall coordination and brain functioning. Multistrain probiotics possess antidiarrheal properties. Diarrhea has become a global health problem. Diarrhea caused by lactose treatment is called osmotic diarrhea [46]. Lactose treatment causes osmotic diarrhea. A high-lactose diet causes persistent diarrhea. According to the results, the rats had diarrhea, as described previously by other researchers [31, 32]. A microbial metabolomics study highlights how multistrain probiotics influence antidiarrheal metabolism. Our understanding of microbial metabolites helps identify targets for improved antidiarrheal treatment.

## Conclusion

We investigated the effects of multistrain probiotics-induced diarrhea on the microbiome and metabolic changes. Our results showed that an inflammatory response, microbiome health, and cecal metabolic restoration are significant and promising alterations found with multistrain probiotics. These results suggest that serotonin, TPH1, and SCFA metabolites represent a promising and practical solution for diarrhea and antidiarrhea recovery. Microbial metabolomics has become mainstream for phenotypic drug screening for antidiarrheal therapeutics.

## Supplementary Information

Below is the link to the electronic supplementary material.

Supplementary file1 (DOCX 631 KB)

## Data Availability

The data in this study are all presented in the article.

## Abbreviations

SD: Sprague-Dawley
AA: Acetic acid
CON: Control
PC: Positive control
SSZ: Sulfasalazine
G: Multistrain probiotics
LC-MS: Liquid chromatography-mass spectrometry
SE: Standard error
PCA: Principal component analysis
PLS-DA: Partial least squares discriminant analysis
GO: Gene ontology
DEG: Differentially expressed genes
ExDEGA: Excel-based differentially expressed gene analysis
