# Supplementary material for: Multistrain Probiotics Alleviate Diarrhea by Modulating Microbiome-Derived Metabolites and Serotonin Pathway
Source: Probiotics Antimicrob Proteins. 2024 Mar 12;17(5):2894–908. doi: 10.1007/s12602-024-10232-4 (PMC12532644; doi:10.1007/s12602-024-10232-4)
Supplement: Supplementary file 1 — Supplementary file1 (DOCX 631 KB) [file 12602_2024_10232_MOESM1_ESM.docx]

Supplementary files for

**Multi-strain probiotics alleviate diarrhea by modulating microbiome-derived metabolites and serotonin pathway**

Jin-Ju Jeong ^1†^, Yoo-Jeong Jin^2†^, Raja Ganesan^1†^, Hee Jin Park^1^, Byeong Hyun Min^1^, Min Kyo Jeong^1^, Sang Jun Yoon^1^, Mi Ran Choi^1^, Satya Priya Sharma^1^, You Jin Jang^2^, Uigi Min^2^, Jong-Hyun Lim^2^, Kyeong Min Na^3^, Jieun Choi^3^, Sang Hak Han^4^, Young Lim Ham^5^, Do Yup Lee^3, *^, Byung-Yong Kim^2, *^, Ki Tae Suk^1, *^

**Author’s affiliations**

^1^ Institute for Liver and Digestive Disease, College of Medicine, Hallym University, Chuncheon, Korea

^2^ R&D Center, Chong Kun Dang Healthcare, Seoul, Republic of Korea

^3^ Department of Agricultural Biotechnology, Center for Food and Bioconvergence, Research Institute of Agricultural and Life Sciences, Seoul National University, Seoul, Korea

^4^ Department of Pathology, College of Medicine, Hallym University, Chuncheon, Republic of Korea

^5^ Department of Nursing Daewon University College Jecheon, Republic of Korea

^†, *^ These authors contributed equally to this work.

* Corresponding author 1: Do Yup Lee

* Corresponding author 2: Byung-Yong Kim

* Corresponding author 3: Ki Tae Suk


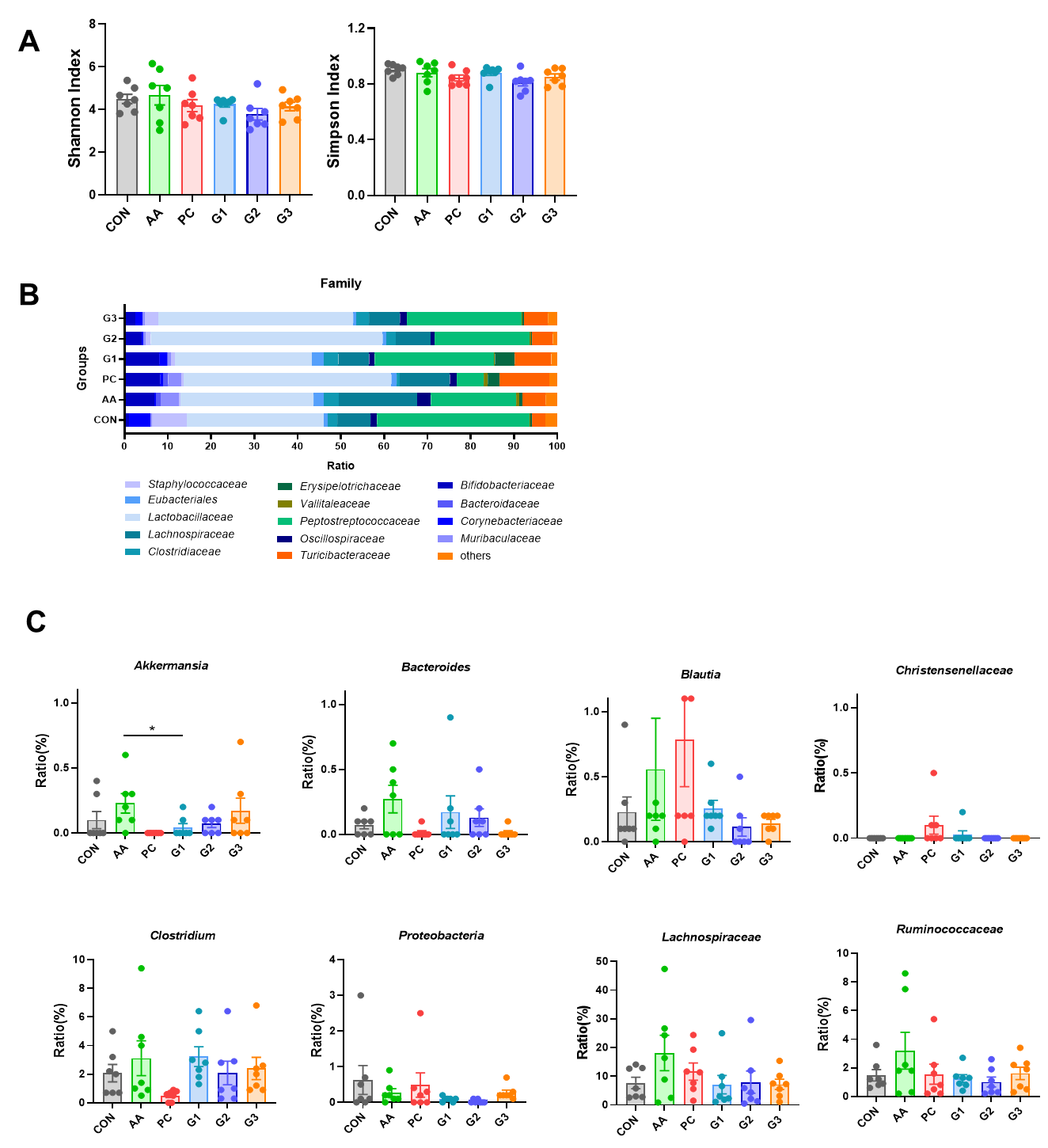


**Fig. S1.** Microbiota. **(A)** Relative bacterial diversity index analysis at Shannon and Simpson levels. **(B)** Family distribution of the number of species that are either elevated or depleted in diarrhea stages compared to the CON. **(C)** Human gut species


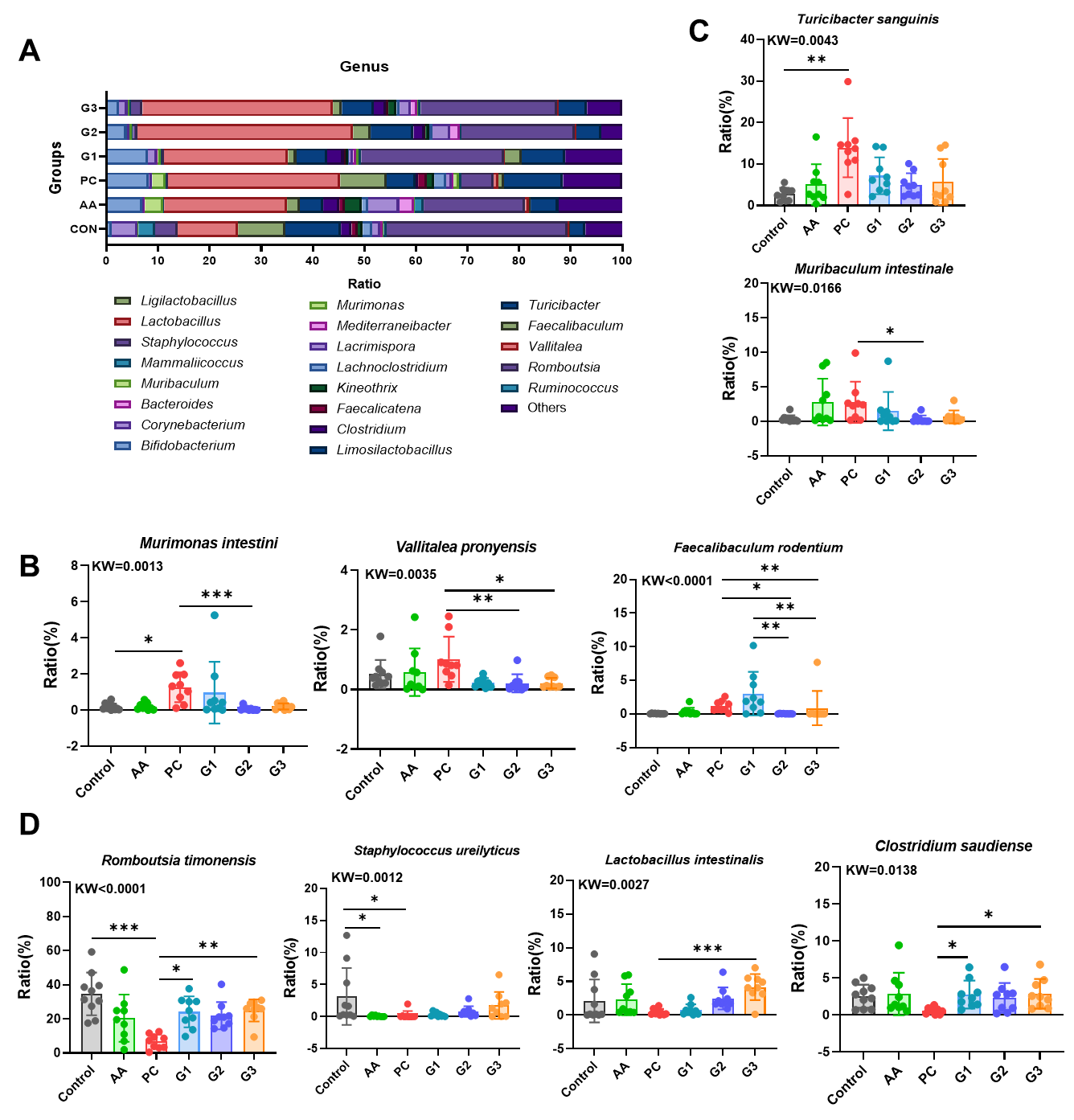


**Fig. S2. (A)** The genus distribution of the number of species that are either elevated or depleted in diarrhea stages compared to the CON. **(B)** We screened the microbial regulation of *Murimonas intestine, Vallitalea pronyensis,* and *Faecalibaculum rodentium.* **(C)** *Turicibacter sanguinis* and *Muribaculum intestinale* were screened. **(D)** *Romboutsia timonensis, staphylococcus spp., Lactobacillus intestinalis,* and *Clostridium sadiense* bacteria were investigated in rats. *, **, and *** represent *p* < 0.05, *p* < 0.01, and *p* < 0.001, respectively. The error bars indicate SEM.


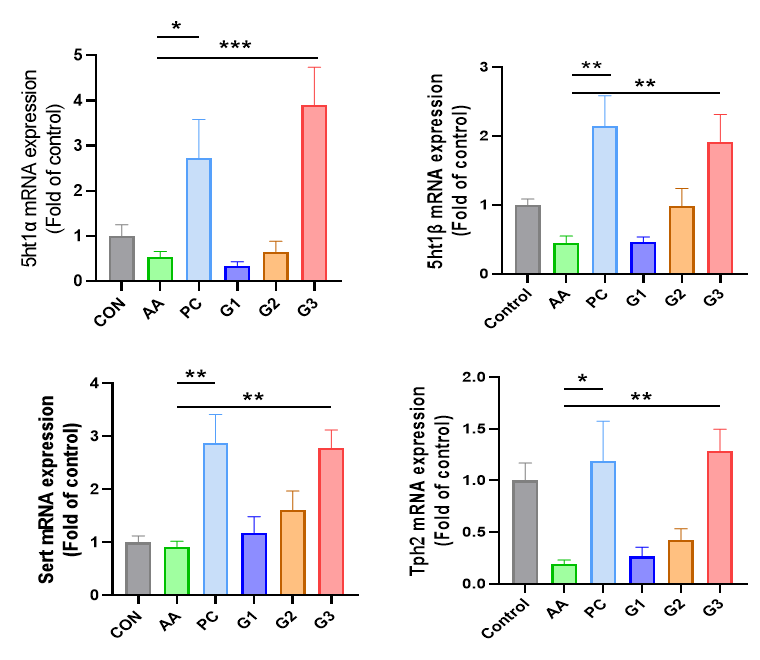


**Fig. S3.** Serotonin relating factors. mRNA level of 5ht1α, 5ht1β, Sert, and Tph2 of colon cells subjected and assessed after the treatment of NC, AA, and G1, G2, and G3 multi-strain probiotics. The probiotics effects and validation has done by qPCR and ELISA methods by multi-strain probiotics has observed. **p* < 0.05, ***p* < 0.01, and ****p* < 0.001. Data values (n = 7) are summarized as mean ± standard error of the mean.

**
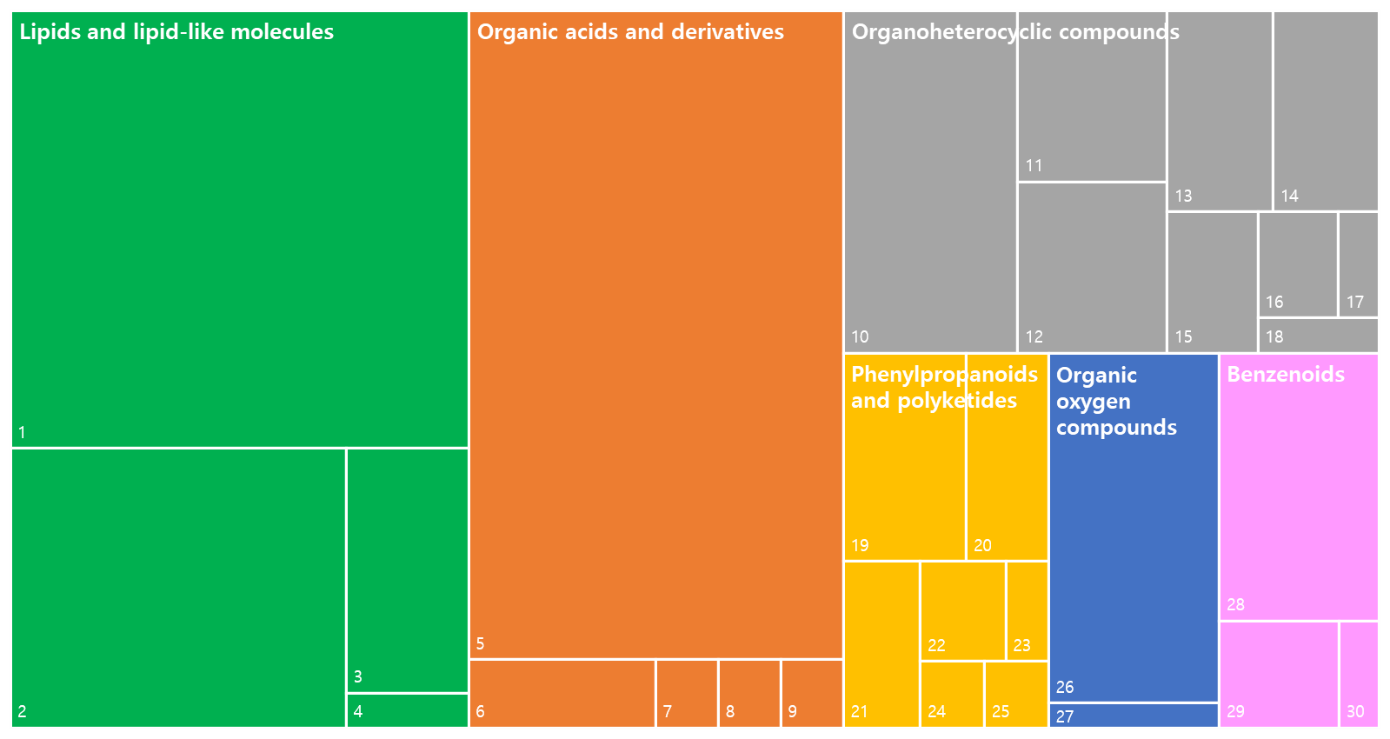
**

**Fig. S4.** Treemap based on chemical taxonomy of metabolites identified in cecum content of diarrhea-induced rats. A total of 230 compounds (92%) were classified into 6 super classes.

**
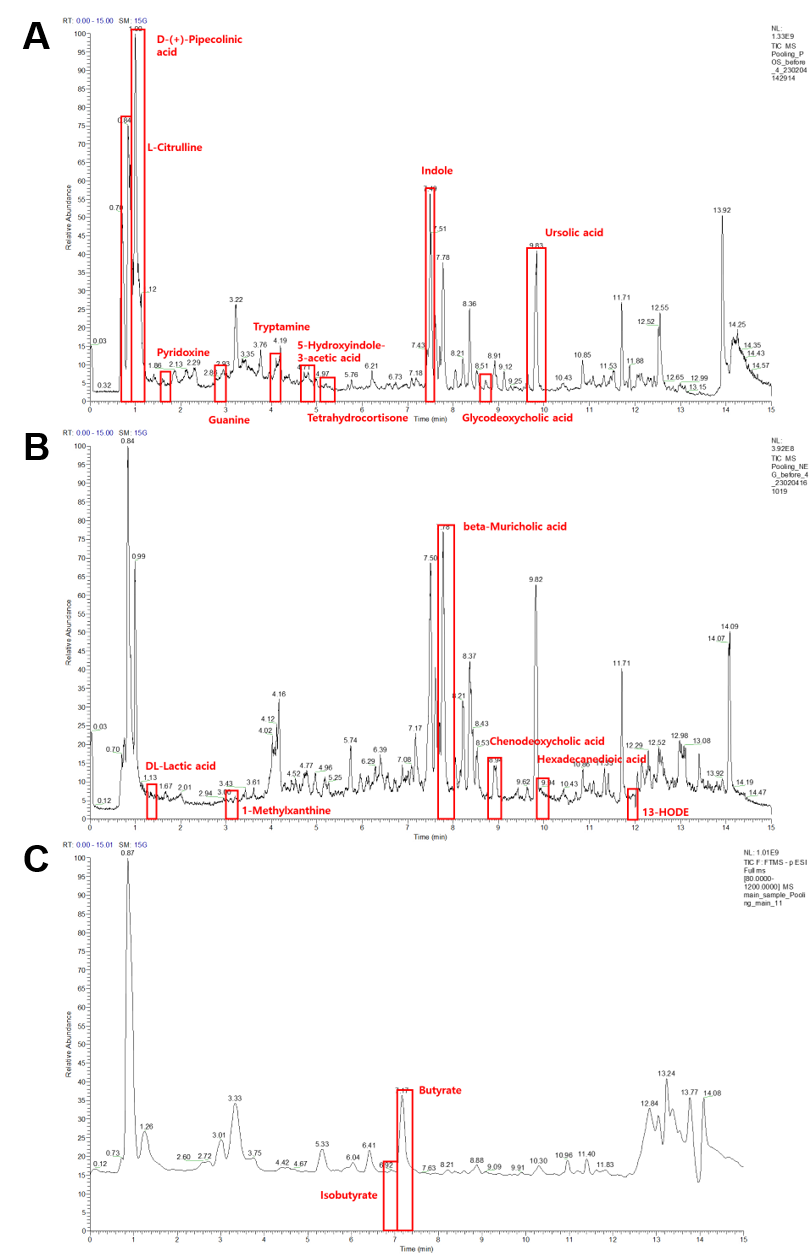
**

**Fig. S5.** Total ion chromatogram (TIC) of pooled sample in untargeted analysis from positive ion mode (A), negative ion mode (B), and derivatized SCFA-targeted analysis (C). Red box indicates metabolites that belong to top 10 of VIP score in multivariate analysis of Fig. 5A or Fig. 5C.

**Table S1.** PCR primers used in this study.

| Gene | Primer | Sequences 5'->3' |
| --- | --- | --- |
| GAPDH | Forward  Reverse | CCATCACCATCTTCCAGGAG  CCTGCTTCACCAACCTTCTTG |
| MUC2 | Forward  Reverse | GATAGGTGGCAGACAGGAGA  GCTGACGAGTGGTTGGTGATTG |
| TPH2 | Forward  Reverse | TAAATACTGGGCCAGGAGAGG  GAAGTGTCTTTGCCGCTTCTC |
| SERT | Forward  Reverse | ATCTCCTAGAACCCTGTAAC  GAAATGGACCTGGAGTATTG |
| 5-HT1A | Forward  Reverse | TCCGACGTGACCTTCAGCTA  GCCAAGGAGCCGATGAGATA |
| 5-HT1B | Forward  Reverse | CCGGCTAACTACCTGATCGC  TATCCGACGACAGCCAGAAG |

**Table S2.** The top 6 superclasses and subordinate classes of the cecal metabolites.

| Superclass | class |
| --- | --- |
| Lipids and lipid-like molecules (77) | Fatty Acyls (47) |
|  | Steroids and steroid derivatives (22) |
|  | Prenol lipids (7) |
|  | Glycerophospholipids (1) |
| Organic acids and derivatives (63) | Carboxylic acids and derivatives (57) |
|  | Hydroxy acids and derivatives (3) |
|  | Carboximidic acids and derivatives (1) |
|  | Keto acids and derivatives (1) |
|  | Organic sulfonic acids and derivatives (1) |
| Organoheterocyclic compounds (43) | Indoles and derivatives (14) |
|  | Diazines (6) |
|  | Imidazopyrimidines (6) |
|  | Pyridines and derivatives (5) |
|  | Quinolines and derivatives (5) |
|  | Azoles (3) |
|  | Tetrapyrroles and derivatives (2) |
|  | Isobenzofurans (1) |
|  | Pyrans (1) |
| Phenylpropanoids and polyketides (18) | Cinnamic acids and derivatives (6) |
|  | Isoflavonoids (4) |
|  | Phenylpropanoic acids (3) |
|  | Linear 1,3-diarylpropanoids (2) |
|  | Cinnamaldehydes (1) |
|  | Coumarins and derivatives (1) |
|  | Flavonoids (1) |
| Organic oxygen compounds (15) | Organooxygen compounds (14) |
|  | Organic oxides (1) |

**Table S3.** Metabolites that differentiate between control, AA, PC, G1, G2 and G3 probiotics in SD rats.

| C vs AA (unique) | HMDB | KEGG | FC | Changes |
| --- | --- | --- | --- | --- |
| Stearic acid | HMDB0000827 | C01530 | 6.583 | ↑ |
| 2-Hydroxyphenylalanine | HMDB0006050 | -- | 4.240 | ↑ |
| Cholic acid | HMDB0000619 | C00695 | 2.986 | ↑ |
| Desoxycortone | HMDB0000016 | C03205 | 2.748 | ↑ |
| Tetrahydrocortisone | HMDB0000903 | -- | 2.475 | ↑ |
| 4-Hydroxybenzaldehyde | HMDB0011718 | C00633 | 2.453 | ↑ |
| 7-Methylguanine | HMDB0000897 | C02242 | 2.359 | ↑ |
| Methylimidazoleacetic acid | HMDB0002820 | C05828 | 2.315 | ↑ |
| L-Aspartic acid | HMDB0000191 | C00049 | 2.176 | ↑ |
| 16-Hydroxyhexadecanoic acid | HMDB0006294 | C18218 | 2.106 | ↑ |
| Stercobilin | HMDB0240259 | -- | 1.985 | ↑ |
| 8-Iso-15-keto-prostaglandin-F2β | -- | -- | 1.882 | ↑ |
| D-(+)-Proline | -- | -- | 1.870 | ↑ |
| Isophorone | HMDB0031195 | C14743 | 1.837 | ↑ |
| 2'-Deoxyuridine | HMDB0000012 | C00526 | 1.786 | ↑ |
| Suberic acid | HMDB0000893 | C08278 | 1.730 | ↑ |
| 4-Pyridoxic acid | HMDB0000017 | C00847 | 1.718 | ↑ |
| Polygodial |  |  | 1.607 | ↑ |
| Xanthine | HMDB0000292 | C00385 | 1.367 | ↑ |
| L-Pyroglutamic acid | HMDB0000267 | C01879 | 1.211 | ↑ |
| β-Alanine | HMDB0000056 | C00099 | 1.204 | ↑ |
| N-Acetylornithine | HMDB0003357 | C00437 | 0.697 | ↓ |
| Pantothenic acid | HMDB0000210 | C00864 | 0.673 | ↓ |
| Acetate | HMDB0000042 | C00033 | 0.633 | ↓ |
| Muramic acid | HMDB0003254 | C06470 | 0.611 | ↓ |
| (+/-)12(13)-DiHOME |  |  | 0.611 | ↓ |
| N-Acetylneuraminic acid | HMDB0000230 | C19910 | 0.602 | ↓ |
| 3-Methoxyphenylacetic acid | HMDB0059969 | -- | 0.572 | ↓ |
| Glycylproline | HMDB0000721 | -- | 0.567 | ↓ |
| 18-HETE | HMDB0006245 | -- | 0.476 | ↓ |
| Ethylmalonic acid | HMDB0000622 | -- | 0.462 | ↓ |
| Nicotinic acid | HMDB0001488 | C00253 | 0.428 | ↓ |
| Thiamine | HMDB0000235 | C00378 | 0.369 | ↓ |
| α-Linolenoyl ethanolamide | HMDB0013624 | C13828 | 0.360 | ↓ |
| Ethyl oleate | HMDB0034451 | C03425 | 0.359 | ↓ |
| Glutaric acid | HMDB0000661 | C00489 | 0.330 | ↓ |
| Tyrosol | HMDB0004284 | C06044 | 0.290 | ↓ |
| Linoleic acid | HMDB0000673 | C01595 | 0.277 | ↓ |
| (+/-)9,10-dihydroxy-12Z-octadecenoic acid | - | - | 0.252 | ↓ |
| α,α-Trehalose | - | - | 0.248 | ↓ |
| Oleoyl ethanolamide | - | - | 0.242 | ↓ |
| Linoleoyl ethanolamide | HMDB0012252 | -- | 0.219 | ↓ |
| 9-Oxo-ODE | HMDB0004669 | C14766 | 0.219 | ↓ |
| Ferulic acid | HMDB0000954 | C01494 | 0.216 | ↓ |
| Pyridoxine | HMDB0000239 | C00314 | 0.181 | ↓ |
| (+/-)9-HODE | -- | -- | 0.175 | ↓ |
| 9-Oxo-10(E),12(E)-octadecadienoic acid | -- | -- | 0.166 | ↓ |
| Indole-3-lactic acid | HMDB0000671 | C02043 | 0.153 | ↓ |
| Isoliquiritigenin | HMDB0037316 | C08650 | 0.152 | ↓ |
| (+/-)9-HpODE | -- | -- | 0.147 | ↓ |
| 13(S)-HOTrE | -- | -- | 0.139 | ↓ |
| Daidzein | HMDB0003312 | C10208 | 0.113 | ↓ |
| Sinapinic acid | HMDB0032616 | C00482 | 0.053 | ↓ |
| 3,4-Dihydroxyphenylpropionic acid | HMDB0000423 | C10447 | 0.025 | ↓ |
| G1 vs AA (unique) |  |  | FC |  |
| N-Isovalerylglycine | -- | -- | 5.016 | ↑ |
| Tiglic acid | HMDB0001470 | C08279 | 4.880 | ↑ |
| 4-Methyl-5-thiazoleethanol | HMDB0032985 | C04294 | 4.696 | ↑ |
| L-Cysteinesulfinic acid | HMDB0000996 | C00606 | 4.500 | ↑ |
| DL-Arginine | -- | -- | 2.561 | ↑ |
| N-Acetylvaline | HMDB0011757 | -- | 2.310 | ↑ |
| L-Valine | HMDB0000883 | C00183 | 2.211 | ↑ |
| Indole-3-acetic acid | HMDB0000197 | C00954 | 2.072 | ↑ |
| Propanate | HMDB0000237 | C00163 | 1.841 | ↑ |
| Kynurenic acid | HMDB0000715 | C01717 | 1.712 | ↑ |
| Hexadecanamide | HMDB0012273 | -- | 1.280 | ↑ |
| Methionine sulfoxide | HMDB0002005 | -- | 1.215 | ↑ |
| Acetylcholine | HMDB0000895 | C01996 | 0.665 | ↓ |
| DL-α-Aminocaprylic acid | HMDB0000991 | -- | 0.557 | ↓ |
| G2 vs NC (unique) |  |  | FC |  |
| Taurine | HMDB0000251 | C00245 | 2.011 | ↑ |
| D-δ-Tocopherol | -- | -- | 2.132 | ↑ |
| Lithocholic acid | HMDB0000717 | C17658 | 1.583 | ↑ |
| Urocanic acid | HMDB0000301 | C00785 | 1.521 | ↑ |
| Adenosine | HMDB0000050 | C00212 | 3.351 | ↑ |
| Cholest-4-en-3-one | HMDB0000921 | C00599 | 3.007 | ↑ |
| 2-Amino-1,3,4-octadecanetriol | -- | -- | 1.849 | ↑ |
| DL-Lysine | -- | -- | 1.577 | ↑ |
| Thymine | HMDB0000262 | C00178 | 0.571 | ↓ |
| Testosterone acetate | HMDB0062780 | C03027 | 0.386 | ↓ |
| G3 vs AA (unique) |  |  | FC |  |
| Glycochenodeoxycholic acid | HMDB0000637 | C05466 | 2.337 | ↑ |
| (+/-)13-HODE | -- | -- | 1.783 | ↑ |
| Azelaic acid | HMDB0000784 | C08261 | 0.577 | ↓ |
| 3-Hydroxybutyric acid | HMDB0000357 | C01089 | 0.410 | ↓ |
| PC vs AA (unique) |  |  | FC |  |
| 1-Methylxanthine | HMDB0010738 | C16358 | 3.270 | ↑ |
| Creatine | HMDB0000064 | C00300 | 1.734 | ↑ |
| Bilirubin | HMDB0000054 | C00486 | 1.718 | ↑ |
| Glycerophospho-N-palmitoyl ethanolamine | -- | -- | 1.643 | ↑ |
| (2R)-2,3-Dihydroxypropanoic acid | -- | -- | 1.630 | ↑ |
| Betaine | HMDB0000043 | C00719 | 1.582 | ↑ |
| Choline | HMDB0000097 | C00114 | 1.557 | ↑ |
| 3,4-Dimethylbenzoic acid | HMDB0002237 | -- | 1.473 | ↑ |
| Salicylic acid | HMDB0001895 | C00805 | 0.710 | ↓ |
| Valeric acid | HMDB0000892 | C00803 | 0.655 | ↓ |
| Pyruvic acid | HMDB0000243 | C00022 | 0.428 | ↓ |
| 3-Coumaric acid | HMDB0001713 | C12621 | 0.423 | ↓ |
| Verrucarol | -- | -- | 0.375 | ↓ |
| (±)-Abscisic acid | -- | -- | 0.348 | ↓ |
| 4-Hydroxybutyric acid (GHB) | -- | -- | 0.301 | ↓ |
| Skatole | HMDB0000466 | C08313 | 0.217 | ↓ |

Metabolites are presented with FC ratio differences. Metabolites affected by AA, PC, G1, G2 and G3 treatments in SD rats.

*Note and Abbreviations:* HMDB, human metabolome database; KEGG, kyoto encyclopedia of genes and genomes; FC, fold changes; AA, negative control; PC, positive control; C, control.
